# 6 H Hydrothermal Synthesis of W-Doped VO_2_(M) for Smart Windows in Tropical Climates

**DOI:** 10.3390/ma19020345

**Published:** 2026-01-15

**Authors:** Natalia Murillo-Quirós, Fernando Alvarado-Hidalgo, Ricardo Starbird-Perez, Erick Castellón, Natalia Hernández-Montero, Hans Bedoya Ramírez, Giovanni Sáenz-Arce, Fernando A. Dittel-Meza, Esteban Avendaño Soto

**Affiliations:** 1 Escuela de Física, Instituto Tecnológico de Costa Rica, Cartago 159-7050, Costa Rica; alex.fernando04@gmail.com; 2 Centro de Investigación en Ciencia e Ingeniería de Materiales (CICIMA), Universidad de Costa Rica, San Pedro 11501-2060, Costa Rica; esteban.avendanosoto@ucr.ac.cr; 3 Programa de Maestría Ingeniería en Dispositivos Médicos, Instituto Tecnológico de Costa Rica, Cartago 159-7050, Costa Rica; 4 Escuela de Química, Instituto Tecnológico de Costa Rica, Cartago 159-7050, Costa Rica; 5 Escuela de Química, Universidad de Costa Rica, San Pedro 11501-2060, Costa Rica; erick.castellon@ucr.ac.cr; 6 Escuela de Ingeniería Química, Universidad de Costa Rica, San Pedro 11501-2060, Costa Rica; natalia.hernandezmontero@ucr.ac.cr (N.H.-M.); hans_0730@hotmail.com (H.B.R.); 7 Departamento de Física, Universidad Nacional, Heredia 86-3000, Costa Rica; gsaenz@una.ac.cr; 8 Centro de Investigaciones de la Escuela de Ciencia e Ingeniería en Materiales (CIEMTEC), Instituto Tecnológico de Costa Rica, Cartago 159-7050, Costa Rica

**Keywords:** vanadium dioxide, VO_2_(M), tungsten doping, hydrothermal synthesis, thermochromic coatings, phase transition, smart windows, tropical climates

## Abstract

**Highlights:**

**What are the main findings?**

- Rapid 6 h hydrothermal synthesis of VO_2_(M)
- pH ≈ 8.5 enables phase stabilization
- W-doping lowers Tc by ~17 °C per wt.%
- Crystallites below 35 nm without annealing
- Powders suitable for dispersion processing

**What are the implications of the main findings?**

- Enables low-cost thermochromic coatings
- Scalable route for smart-window materials
- Facilitates integration into polymer films

**Abstract:**

Thermochromic smart windows are a promising technology to reduce energy consumption in buildings, particularly in tropical regions where cooling demands are high. Vanadium dioxide (VO_2_) is the most studied thermochromic material due to its reversible semiconductor-to-metal transition near 68 °C. Conventional synthesis routes require long reaction times and post-annealing steps. In this work, we report a rapid hydrothermal synthesis of monoclinic VO_2_(M) and tungsten-doped VO_2_(M) powders obtained within only 6 h at 270 °C, using vanadyl sulfate as precursor and controlled precipitation at pH ≈ 8.5. Differential scanning calorimetry confirmed the reversible transition at 59 °C for the undoped VO_2_, with a hysteresis of 18 °C, while tungsten doping reduced the transition temperature by ~17 °C per wt.% of W. X-ray diffraction verified the monoclinic phase with minor traces of VO_2_(B), a non-thermochromic polymorph of VO_2_, and microstructural analysis revealed crystallite sizes below 35 nm. Electron microscopy and dynamic light scattering confirmed particle sizes suitable for dispersion in polymeric matrices. This approach significantly reduces synthesis time compared to typical hydrothermal methods requiring 20–48 h and avoids further annealing. The resulting powders provide a low-cost and scalable route for fabricating thermochromic coatings with transition temperatures closer to ambient conditions, making them relevant for smart-window applications in tropical climates, where lower transition temperatures are generally regarded as beneficial.

## 1. Introduction

Thermochromic smart coatings have gained increasing attention as a strategy to improve the energy performance of buildings [1,2]. Among building components, windows represent one of the main sources of energy loss, as they simultaneously permit heat gain and heat dissipation [3,4]. Thermochromic smart windows address this limitation by dynamically regulating near-infrared radiation. Their optical response changes from a transparent to a reflective state as the ambient temperature increases, without the need for external stimuli, thereby contributing to reduced energy consumption [2,5].

Vanadium dioxide (VO_2_) is regarded as one of the most extensively investigated thermochromic material [6,7]. It has been incorporated into a wide range of architectures, including thin films [8,9], nanocomposites [10], micro-composites, and advanced engineered structures such as grids or biomimetic designs [11]. VO_2_ undergoes a reversible first-order phase transition from a monoclinic semiconducting phase (M) to a metallic rutile-type tetragonal phase (R) when the temperature reaches approximately 68 °C [12,13]. This transition is accompanied by a pronounced decrease in electrical resistivity and a substantial increase in infrared reflectance, while the optical response in the visible range remains only weakly affected [14] Although several crystalline phases of VO_2_ have been reported, only the M and R phases exhibit a fully reversible semiconductor–metal transition [15].

One effective strategy to shift the transition temperature toward ambient conditions is the incorporation of dopants into the VO_2_ lattice. In particular, tungsten (W) doping has been shown to produce one of the strongest reductions in the transition temperature, with experimentally reported rates of approximately 20–26 °C per wt.% [16]. This tunability is especially relevant for applications in thermochromic windows operating under warm climatic conditions.

Hydrothermal synthesis is widely employed for the preparation of metal oxides and is based on chemical reactions occurring in aqueous environments under elevated temperature and pressure. This approach is commonly used for the synthesis of VO_2_ nanoparticles [17,18,19], typically requiring temperatures close to 240 °C [20] and reaction times ranging from 20 to 48 h [20,21]. Owing to the multiple stable oxidation states of vanadium, achieving phase-pure VO_2_ remains challenging. Key parameters such as temperature, reaction time, pressure, precursor feeding rate, reducing agents, and solution chemistry must be carefully controlled to avoid the formation of undesired vanadium oxide phases [7,22,23].

Previous studies report that vanadium oxides can be obtained via hydrothermal routes through different mechanisms, including the reduction in pentavalent vanadium precursors (e.g., V_2_O_5_ or NH_4_VO_3_) [24,25] using reducing agents, as well as homogeneous precipitation starting from VOSO_4_ under relatively mild hydrothermal conditions [26]. In recent years, one-step hydrothermal synthesis of monoclinic VO_2_(M) powders has attracted growing interest, as it eliminates the need for a secondary heat-treatment step and helps suppress particle aggregation and excessive grain growth [27].

Tropical regions are characterized by high ambient temperatures throughout the year, which are further increasing as a consequence of climate change [28,29]. Experimental and simulation studies on thermochromic windows designed for hot climates indicate that these systems can effectively reduce energy loads even in architectural elements such as skylights and sunroofs [30]. Furthermore, it has been demonstrated that lowering the phase transition temperature toward room temperature leads to enhanced energy savings in warm climates compared to regions dominated by cold weather conditions [14,31,32,33].

In this work, we report a rapid one-step hydrothermal precipitation route for the synthesis of doped and undoped VO_2_(M) particles, achieved within 6 h and without the need for post-annealing treatments. The resulting materials are suitable for integration into thermochromic smart-window systems, where they can contribute to reduced energy consumption by selectively modulating infrared radiation while preserving visible light transmission, which is essential for indoor comfort and human well-being.

## 2. Materials and Methods

### 2.1. Synthesis

Vanadium(IV) oxysulfate (VOSO_4_), ammonium paratungsten ((NH_4_)_10_W_12_O_41_·5H_2_O), ammonia (NH_3_), and xylene were purchased from Sigma-Aldrich (San José, Costa Rica). All the reagents used were ACS grade. Deionized water was used in all the sample preparations.

The synthesis is based on a hydrothermal-assisted homogeneous precipitation approach [19], using the inexpensive and low-toxic VOSO_4_ as a raw material to produce VO_2_(M) phase. The reported W concentrations correspond to nominal wt.% values introduced during synthesis. For the synthesis, 1.00 g of VOSO_4_ was dissolved in 30 mL of deionized water. Tungsten-doped samples were prepared by adding 14.6 or 29.2 mg of (NH_4_)_10_W_12_O_41_·5H_2_O, corresponding to nominal W contents of 0.5 and 1.0 wt.%, respectively, while an undoped sample was prepared as a reference. Apart from the initial precursor composition, all samples were synthesized following the same procedure.

The formed dispersion was continuously stirred at a temperature of 60 °C under argon bubbling until a translucent blue solution with a pH value of approximately 2.5 was obtained. Subsequently, an aqueous ammonia solution (NH_3_, 23%) was added dropwise until the pH reached 8.5 (approximately 2.5 mL), leading to the formation of a pink–grayish precipitate. Temperature and stirring were maintained throughout the precipitation process. The precipitate was collected by centrifugation and washed three times with deionized water, discarding the supernatant after each cycle.

After washing, the precipitate was transferred to a custom madeTeflon liner containing 20 mL of deionized water. The Teflon container (nominal volume 30 mL) was purged with argon prior to sealing and subsequently placed inside a stainless-steel autoclave. The hydrothermal treatment was carried out at 270 °C for 6 h. The external furnace used did not operate under controlled atmosphere. After completion of the reaction, the autoclave was allowed to cool naturally to room temperature by heat exchange with the ambient environment.

**Figure 1:**
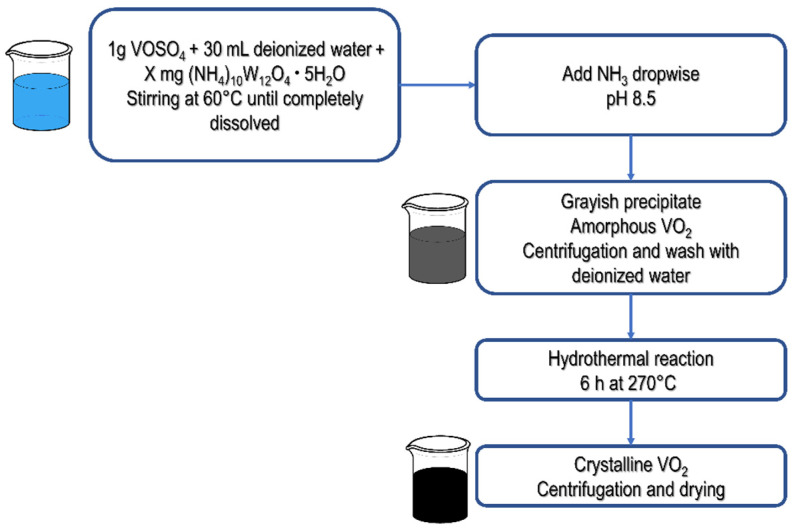
Schematic representation of the hydrothermal-assisted homogeneous precipitation route used for the synthesis of undoped and W-doped VO_2_(M) powders. Vanadium(IV) oxysulfate (VOSO_4_) and ammonium paratungstate (NH_4_)_10_W_12_O_41_·5H_2_O are dissolved in water under stirring and inert atmosphere, followed by dropwise addition of ammonia (NH_3_) to induce precipitation. The precipitate is collected, washed, and transferred to a Teflon^®^ liner, sealed in a stainless-steel autoclave, and hydrothermally treated at 270 °C for 6 h to obtain VO_2_(M). Arrows indicate the sequence of the synthesis steps. The parameter X corresponds to the amount of ammonium paratungstate added (X = 0, 14.6 mg, or 29.2 mg), yielding undoped, 0.5 wt.% W-doped, and 1.0 wt.% W-doped samples, respectively.

The resulting black powder was recovered by centrifugation, washed with deionized water, and dried under a mechanical vacuum at 60 °C for 8 h. The schematic synthesis procedure is shown in Figure 1.

### 2.2. Characterization

#### 2.2.1. Thermal Characterization

The thermal characterization was carried using a DSC250 (TA Instruments, New Castle, DE, USA), using a heating and cooling rate of 10 °C min^−1^ in inert atmosphere with a flow rate of 50 mL min^−1^ of nitrogen (UHP) (Praxair, San José, Costa Rica), in a temperature range from 0 to 100 °C. The reversible first-order phase transition was measured by extrapolations of the baselines to a selected temperature range at which the sharp enthalpy signal was detected. The thermogravimetric study (TG) of the samples was conducted on a SDT-600 thermal analyzer (TA Instruments, New Castle, DE, USA) under dry air (Praxair, San José, Costa Rica) flow in the range of −50–550 °C, with a heating rate of 10 °C min^−1^.

#### 2.2.2. Electron Microscopy

The samples were collected on a metal grid for transmission electron microscopy (TEM) observation. The images were obtained in a TEM (JEM-2011, Jeol Tokyo, Japan) at 200 kV. The samples were analyzed using a scanning electron microscopy SEM Cube-II (EM-Crafts, Hanam, Korea), at 10 kV. EDS mapping was performed using a Xplore 30 mm (Oxford Instruments, Abingdon, UK) and the images were analyzed using the AZtec software (version 6.0, Oxford Instruments).

#### 2.2.3. Dynamic Light Scattering (DLS)

The hydrodynamic diameter of the dispersions was measured using a Nano ZS Zetasizer instrument (Malvern Panalytical Ltd., Worcester, UK). All measurements were performed at 25 °C at 173° angle in xylene, after high-power ultrasound treatment of the samples using a Sonifier QSonica (Ultrasonic Corporation, Newtown, CT, USA).

#### 2.2.4. X-Ray Diffraction (XRD)

X-ray diffraction was carried out in a diffractometer EMPYREAN (Malvern Panalytical Ltd., Worcester, UK). All the analyses were performed using a Cu K-alpha: 1.54060 Å source, 45 kV. Results were compared to a database using the software Highscore Plus 3.0.5 of Malvern (Malvern Panalytical Ltd., Worcester, UK). The employed scan was a Gonio type, using a step of 0.0130° 2Theta and 29.07 s step^−1^. The Rietveld refinement with Gsas II software (version #5798) were performed for crystal structure parameter calculations of VO_2_ (M) phase in the samples. Calculations by Williamson Method with software Highscore Plus 3.0.5 of Malvern (Malvern Panalytical Ltd., Worcester, UK).

## 3. Results and Discussion

Conventional hydrothermal routes for the one-step preparation of VO_2_(M) typically involve long reaction times, commonly ranging from 20 to 48 h [24,27]. In this work, the synthesis parameters were systematically evaluated to identify the minimum conditions required for stabilizing the monoclinic VO_2_(M) phase. Based on the analysis of the reversible enthalpy associated with the semiconductor–metal phase transition, a reaction time of 6 h at 270 °C was identified as sufficient to obtain VO_2_(M), enabling a substantial reduction in synthesis time compared to conventional approaches.

The experimental results further show that strict control of both the solution pH and the reaction atmosphere is essential for the successful synthesis of W-doped VO_2_(M). Acidic conditions, as well as the presence of oxygen, were found to promote the formation of non-thermochromic vanadium oxide phases. Previous studies have indicated that maintaining the pH within a neutral to slightly basic range favors the controlled nucleation and growth of nanoscale VO_2_ particles [20,26,34,35]. In line with these observations, a gradual increase in pH was implemented in the present synthesis by the controlled addition of NH_3_, until a final pH value of 8.5 was reached, enabling the formation of particles.

**Figure 2:**
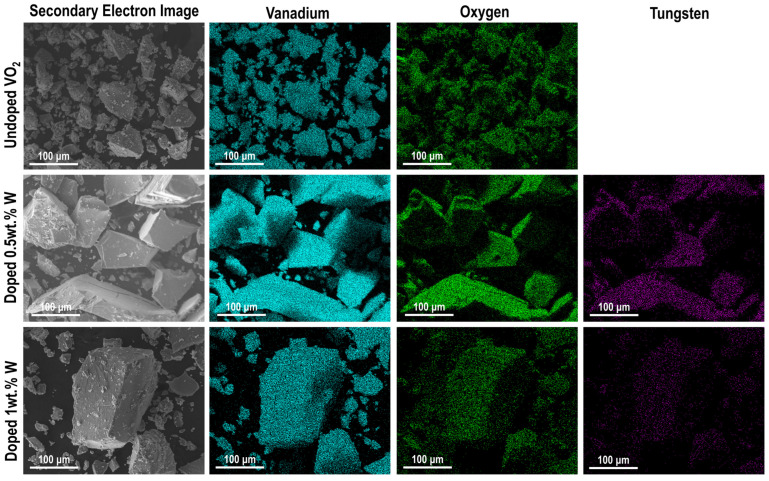
Elemental mapping of undoped and W-doped VO_2_ obtained by energy dispersive spectroscopy on the scanning electron microscope (EDS-SEM). EDS map shows detection of vanadium (cyan), oxygen (green), and tungsten (purple).

The elemental composition of the samples was evaluated by energy-dispersive X-ray spectroscopy (EDS) and mapping the vanadium, tungsten, and oxygen emission lines (see Figure 2). The tungsten emission lines (L_α_ = 8.398 keV and M_α_ = 1.775 keV) were observed in the W-doped samples and its effect in the thermal behavior was confirmed using differential scanning calorimetry.

**Figure 3:**
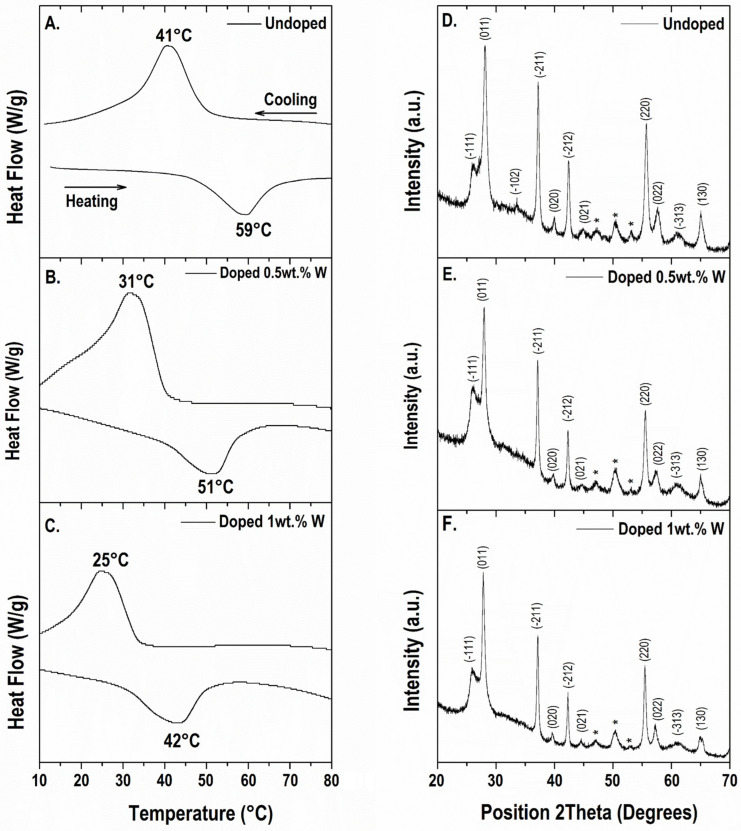
Differential scanning calorimetry of (**A**) Undoped VO_2_, (**B**) Doped with 0.5 wt.% W, (**C**) Doped with 1 wt.% W, using a scan rate of 10 °C min^−1^ in inert atmosphere. X-Ray Diffraction analysis of hydrothermal-produced VO_2_ samples of (**D**) Undoped, (**E**) Doped with 0.5 wt.% W, (**F**) Doped with 1 wt.% W. The asterisks (*) indicate weak diffraction peaks that cannot be assigned to VO_2_(M) reflections and may originate from minor secondary phases.

Differential Scanning Calorimetry (DSC) showed the reversible phase transition by measuring the heat capacity and the differences in the enthalpy of the two phases under specific conditions. DSC analyses are shown in Figure 3A–C. These results confirmed the reversible phase transition of the obtained hydrothermal VO_2_ powders, with a first order thermal transition at 59 °C with 18 °C of hysteresis for the undoped sample whereas the thermal hysteresis varied from 19 to 16 °C for 0.5 and 1.0 wt.%, respectively. The phase transition enthalpy of the undoped VO_2_ sample was found close to 16.0 J g^−1^ for heating as well as for the cooling process. This value is lower compared to those found for hydrothermal VO_2_ [24,36], and as expected, decreases for the doped samples [37]. These enthalpy differences may be attributed to variations on the crystallinity degree of the powdered sample (confirmed by XRD), as well as particle sizes. Transition temperature of doped-VO_2_ with 0.5 as well as 1 wt.% tungsten decreased nearly to 17 °C per tungsten wt.%, this result is similar to those found in the literature for hydrothermally obtained doped-VO_2_ [26,38], thus confirming the presence of tungsten in the VO_2_ matrix and its role on tuning the transition temperatures for hydrothermal VO_2_ [4,39].

The crystalline structure of the synthesized VO_2_ samples was studied by X-ray powder diffraction (XRD) analysis. In the synthesis of hydrothermal VO_2_, several metastable phases may be formed [40]. Results shown in Figure 3D–F confirmed that most signals can be indexed as the monoclinic VO_2_(M) phase according to ICCD 04-003-2035 and references found in the literature [35,36,41,42]. Phase confirmation of the oxides in the samples via XRD is complex to define because of the similarities between VO_2_(M) (P2_1/c_), VO_2_(R) (P4_2/mnm_) phases [43], the subtle difference between these phases at (−111) and (−102) diffraction planes could be masked because of the amorphous contribution. However, three diffraction peaks in the 2theta range of 47–52° were not assigned to monoclinic VO_2_. By comparing these peaks with analyses found in the literature, they are most likely to be part of a fraction of metastable VO_2_(B), formed as part of the hydrothermal synthesis [40]. The low signal-baseline ratio suggests the presence of amorphous material in the sample, supporting the calorimetric results. However, the matching of the diffraction peaks with card ICCD 04-003-2035 VO_2_ (M) confirmed a rapid hydrothermal synthesis within 6 h. The Rietveld refinement for crystal structure parameter calculations of VO_2_(M) phase [44] were performed in the samples, this information is shown in Table A1.

The presence of tungsten as a dopant in the samples is difficult to verify by direct assignment of XRD peaks, as observed when comparing E and F with Figure 3D. This behavior can be attributed to the low tungsten content relative to VO_2_, which likely masks any distinct diffraction signal associated with tungsten, as previously reported in similar studies [26,35].

**Figure 4:**
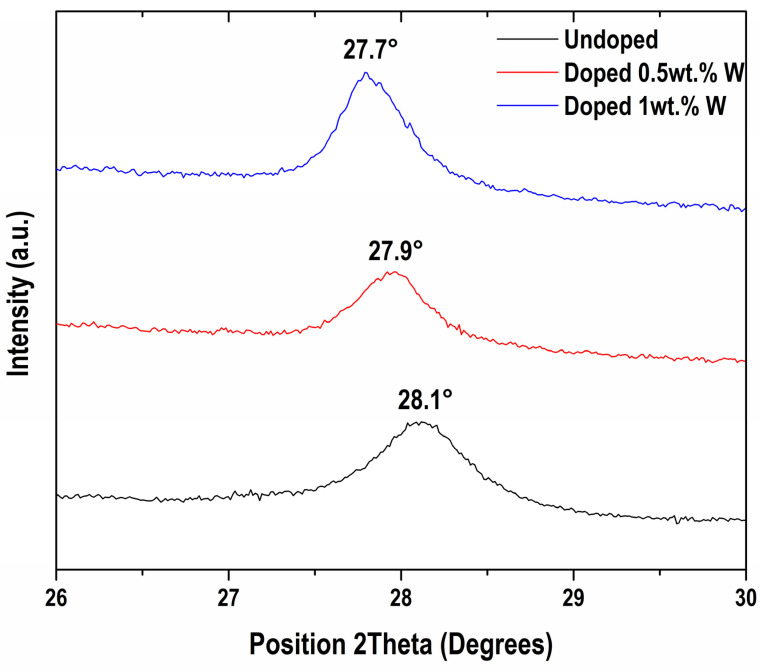
Tungsten doped samples showed a shift in the main diffraction peak towards smaller angles.

Nevertheless, the influence of W-doping on the VO_2_ microstructure is evident in Figure 4, where the main (011) reflection exhibits a gradual shift toward lower diffraction angles as the W content increases, approximately 0.4° in 2Theta per wt.% of W. Williamson–Hall analysis suggests crystallite sizes of 24.2, 24.8, and 34.8 nm for undoped VO_2_(M), VO_2_ doped with 0.5 wt.% W, and VO_2_ doped with 1 wt.% W, respectively. In addition to changes in crystallite size, the observed peak shift may also be influenced by lattice strain induced by tungsten incorporation, as local structural distortions can alter diffraction conditions beyond a simple increase in interplanar spacing [45].

This behavior has been previously reported [46,47] and is commonly attributed to the substitution of larger W^6+^ ions for V^4+^ ions within the VO_2_ lattice, leading to an expansion of the interplanar distance [26]. The effect of tungsten doping is further evidenced by an approximate 0.05° broadening of the (011) peak (Figure 4), which is associated with non-uniform crystallite strain [48]. Additionally, differences in ionic radius have been reported to influence the V–O bonding environment, resulting in higher binding energies between vanadium and oxygen [35].

**Figure A1:**
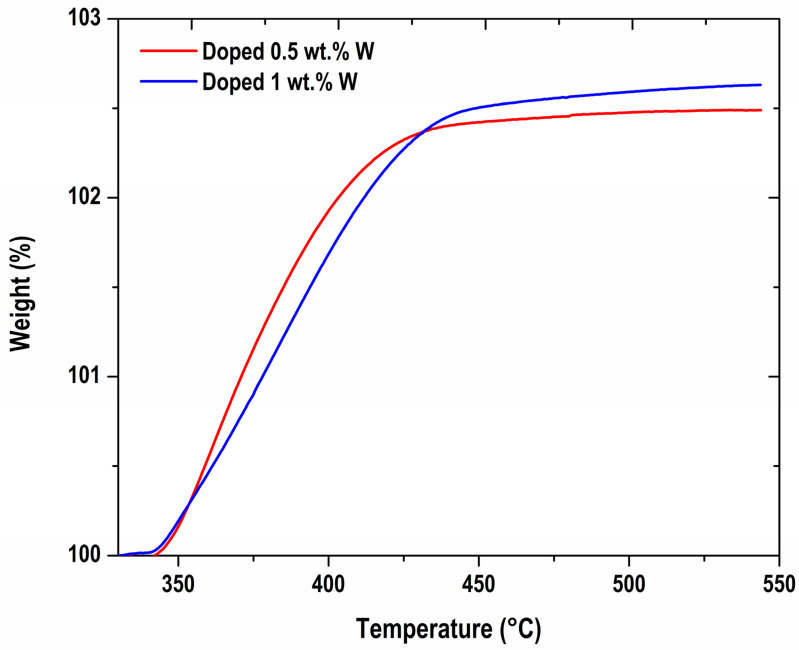
TGA analyses of synthesized of undoped and W-doped VO_2_ and in the air atmosphere.

To understand the stability of the W-doped and undoped VO_2_, the thermogravimetric analysis was conducted in a synthetic air atmosphere, as shown in Appendix A Figure A1. The powder samples were heated in the oxidant atmosphere showing a mass gain due to oxidation, beginning near 350 °C and finishing at 550 °C with a weight gain of ca. 3.0%. The weight gain values of VO_2_ (M) correspond well to the oxidation of the bulk VO_2_ to V_2_O_5_ [49].

**Figure 5:**
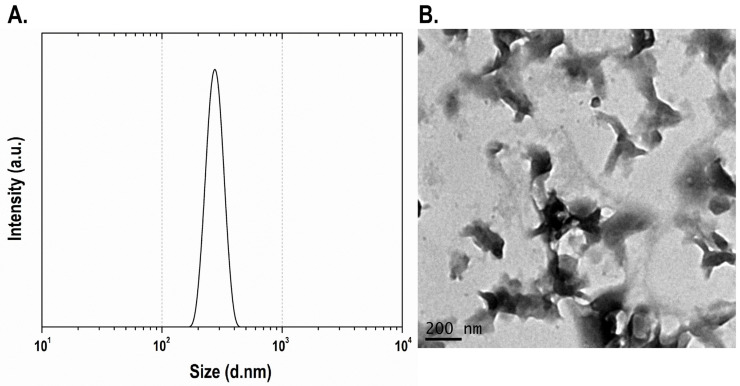
(**A**) Dynamic light scattering on the VO_2_(M) sample in xylene, (**B**) Transmission electron microscopy images of the obtained VO_2_(M) particles.

Finally, for dynamic light scattering (DLS) measurements the bulk VO_2_ was dispersed in xylene using mechanical exfoliation. The powders were dispersed in xylene at a concentration of 1% (*m*/*v*). Prior to measurement, the dispersions were sonicated for 1 min at 30% of the maximum amplitude to ensure adequate deagglomeration. The analysis shows agglomerates with particle sizes close to 200 nm in diameter, as shown in Figure 5A.

Then, the particles were evaluated by transmission electron microscopy (TEM) analyses (Figure 5B) confirming the formation of microcrystals. The bulk material exfoliation into nanocrystals (larger than 35 nm) using mechanical techniques must be further studied, in order to tailor these thermochromic materials inside a polymeric matrix. The undoped and W-doped VO_2_ exfoliation is a promising way to tune their properties for the fabrication of low cost thermochromic devices.

## 4. Conclusions

We demonstrated a fast and low-toxicity hydrothermal-assisted precipitation route for producing thermochromic VO_2_(M) within only 6 h, without any post-annealing.

A slightly basic precipitation environment (pH ≈ 8.5) was identified as a key requirement for stabilizing the monoclinic VO_2_(M) phase. The method also enabled effective W incorporation into the VO_2_ lattice, resulting in a systematic decrease in the phase-transition temperature by approximately 17 °C per wt.% W and crystallite sizes below 35 nm. These outcomes highlight that the proposed route not only simplifies VO_2_ synthesis but also provides intrinsic control over its thermochromic properties.

The undoped and W-doped powders obtained here represent promising starting materials for smart-window coatings, pending further optimization of particle-size distribution and dispersion in polymeric matrices. Overall, this work provides a practical and scalable synthesis route that supports the development of cost-effective thermochromic VO_2_-based materials, which may be of interest for energy-efficient building applications in tropical climates.

## Data Availability

The original contributions presented in the study are included in the article, further inquiries can be directed to the corresponding author.

## Appendix A

### Appendix A.1. Rietveld Refinement Structure Analysis

**Table A1:** Rietveld refinement structure parameters for VO_2_(M) in the samples.

**Sample**	**Cell Parameters**				**Atomic Coordinates**								
	**a** **(Å)**	**b** **(Å)**	**c** **(Å)**	**β** **(°)**	**V1**			**O_2_**			**O_3_**		
					**x**	**y**	**z**	**x**	**y**	**z**	**x**	**y**	**z**
VO_2_ undoped	5.7515	4.5395	5.3814	122.650	0.24102	0.97891	0.02701	0.10863	0.20946	0.02701	0.39959	0.70558	0.29970
VO_2_ Doped 0.5 wt.% W	5.7470	4.5395	5.3808	122.569	0.23976	0.97229	0.02721	0.09393	0.20676	0.21005	0.38503	0.70717	0.29030
VO_2_ Doped 1 wt.% W	5.7250	4.5425	5.3563	122.172	0.24499	0.97312	0.02398	0.09420	0.20719	0.20478	0.39127	0.69770	0.28858
